# The Effects of the Civility, Respect, and Engagement in the Workplace (CREW) Program on Social Climate and Work Engagement in a Psychiatric Ward in Japan: A Pilot Study

**DOI:** 10.3390/nursrep11020031

**Published:** 2021-05-01

**Authors:** Utako Sawada, Akihito Shimazu, Norito Kawakami, Yuki Miyamoto, Lisa Speigel, Michael P. Leiter

**Affiliations:** 1 Department of Psychiatric Nursing, Graduate School of Medicine, The University of Tokyo, 7-3-1 Hongo, Bunkyo-ku, Tokyo 113-0033, Japan; yyuki-tky@umin.ac.jp; 2 Faculty of Policy Management, Keio University, 5322 Endo, Fujisawa, Kanagawa 252-0882, Japan; ashimazu-tky@umin.ac.jp; 3 Department of Mental Health, Graduate School of Medicine, The University of Tokyo, 7-3-1 Hongo, Bunkyo-ku, Tokyo 113-0033, Japan; norito@m.u-tokyo.ac.jp; 4 Technology Services, Beveridge Arts Centre, Acadia University, Wolfville, NS B4P 2R6, Canada; lisa.speigel@acadiau.ca; 5 School of Psychology, Deakin University, Geelong 3217, Australia; michael.leiter@acadiau.ca; 6 Psychology Department, Acadia University, Wolfville, NS B4P 2R6, Canada

**Keywords:** civility, respect, work engagement, social climate, psychiatric ward, CREW

## Abstract

Background: Good social climate and high work engagement are important factors affecting outcomes in healthcare settings. This study observed the effects of a program called Civility, Respect, and Engagement in the Workplace (CREW) on social climate and staff work engagement in a psychiatric ward of a Japanese hospital. Methods: The program comprised 18 sessions installed over six months, with each session lasting 30-min. Participation in the program was recommended to all staff members at the ward, including nurses, medical doctors, and others, but it was not mandatory. A serial cross-sectional study collected data at four time-points. Nurses (n = 17 to 22), medical doctors (n = 9 to 13), and others (n = 6 to 10) participated in each survey. The analysis of variance was used to evaluate the changes in the following dependent variables, the Essen climate evaluation schema (EssenCES), the CREW civility scale, and the Utrecht work engagement scale (UWES) over time. Result: We found no significant effects. The effect size (Cohen’s d) for EssenCES was 0.35 from baseline to post-installation for all staff members. Effect sizes for EssenCES for medical doctors and UWES for nurses were 0.79 and 0.56, respectively, from baseline to post-program. Conclusions: Differences in social climate and work engagement among Japanese healthcare workers between the baseline and post-installation of the CREW program were non-significant.

## 1. Introduction

The psychosocial work environment, comprising workplace social support and social climate [1], has been associated with workers’ mental health [2,3]. The social climate is generated and fostered by human relations, interaction, and communication. In healthcare settings, the social climate affects health, job satisfaction, and wellbeing among nurses [4], decreases turnover rate among physicians [5] and increases job satisfaction among healthcare professionals [6]. Particularly, a social climate focused on workplace social support and welfare is related to high work engagement among physicians and nurses [7]. Other evidence indicates that social climate influences the engagement of nurses in medical/surgical units, intensive care units, obstetrics, operating/recovery rooms, pediatrics, and psychiatry and mental health nurses [8,9]. Work engagement is a positive and fulfilling psychological work-related state [10,11] that is positively associated with workers’ physical and mental health [12,13,14]. The social climate also affects patients. Various studies in psychiatric settings have indicated the importance of social climate, particularly for inpatients, patient satisfaction [15]; patient treatment outcomes, such as treatment drop-out; length of stay; and wellbeing [16,17,18,19,20]. Therefore, healthcare settings’ social climate should be constantly evaluated and modified to improve healthcare staff and patient outcomes.

Some interventions have been designed to improve workplace climate. It is known that effective interventions involve acting simultaneously at different levels, affecting the structure and organization of work and relations between workers. For example, different interventions were reported that improve the working conditions, including the structure, organization, and relationships between workers in psychiatric units [21].

On the other hand, programs, such as Civility, Respect, and Engagement in the Workplace (CREW), focus on enhancing workers’ relationships and communication to improve the work environment. This organizational intervention program was first developed and used in Veterans Hospital Administration settings in the United States in 2005 [22]. This program specifically targets workplace civility norms, and the program’s goal is to improve workplace climate through civil and respectful interactions, communication, and teamwork among colleagues. In previous studies, the CREW program has shown a meaningful improvement in civility level in the workplace, work-related outcomes (e.g., job satisfaction, job resources, support, burnout, trust in management, absenteeism, organizational commitment, and workplace incivility) [22,23,24,25,26], and mental health symptoms [26]. In general, poor communication between healthcare professionals is an important factor that reduces patient safety and patient care quality [27,28,29], and civility must be followed for effective communication [30]. Therefore, civility in communication among healthcare professionals should be promoted. The CREW program operates through a series of sessions (e.g., dialogs, exercises, and role-play designed to include relationship issues on the workgroup’s problem-solving agenda) implemented for about six months [22,23]. These dialogs and discussions allow an organization to address job interactions through collective awareness [23,24]. Typically, all employees who work in a team/department are invited to participate in the program. However, participation is not mandatory. The approach and detailed contents vary according to workplace issues in particular units involved [22]. The CREW program was developed as an evidence-based solution, and it has become more widely known due to its steadily increasing participation rates and the accumulated experience of implementation in Veterans Administration settings (e.g., human resources, call center, and police) and Canadian hospitals [23,24,25]. Although it is necessary to hold regular CREW sessions, no new equipment or advanced techniques are required; therefore, the CREW program is a low-cost, low-tech initiative [24]. It is also a flexible program that can be custom-tailored to suit the workplace and grow and adapt gradually [24].

Civility, which describes respectful communication behaviors between people, is an essential concept of the CREW program and an important part of the organizational culture and positive psychological climate [25,31,32,33,34]. The CREW program incorporates civility as it consciously cultivates an awareness of one’s effects on interpersonal relations at the workplace [24]. The civil norm improves organizational climate, workplace civility, mutual respect [22,31], workplace resources, and employees’ wellbeing [24,31]. Civility helps maintain norms for mutual respect [31], and these norms encourage respectful behaviors among employees, promote cooperation, facilitate communication, and positively affect the overall work environment [35]. For example, previous studies have indicated that high civility climate is significantly related to high care performance among nurses [36,37]. It is considered important for individuals, organizations, and patients to foster civility in nursing education and practice [38,39]. While previous CREW program studies have evaluated the effect of civility social climate on work-related outcomes [23,24,25], no study has explored work engagement. In addition, no studies have investigated social climate and work engagement among Japanese medical staff or programs to improve both the work social climate and work engagement simultaneously. Furthermore, the CREW program has been widely implemented in the United States [24] and Canada [25], but not in Japan.

This study aimed to evaluate the effects of a workplace program called Civility, Respect, and Engagement in the Workplace (CREW) on the social climate and work engagement in a psychiatric ward at a Japanese hospital. We hypothesized that the social climate and work engagement assessed by the staff members working in a Japanese hospital would be better after the CREW program installation compared to a time-point before the program was installed. The effect of the CREW program on work engagement has never been reported. This is the first study investigating the effects of the CREW program on social climate and work engagement among healthcare staff in a Japanese hospital.

## 2. Methods

This pretest/posttest study administered a survey at four time-points to observe changes in hospital ward social climate and work engagement of all the staff members working in the ward before and after the CREW program installation at a psychiatric ward of a Japanese university teaching hospital. Approximately seventy staff members from moderately diverse professional backgrounds (e.g., nurses, medical doctors, psychiatric social workers, occupational therapists, clinical psychologists, pharmacists, cleaning staff, nursing assistants, and medical clerks) participated in the study. The number of staff members and staff composition differed over time because of monthly personnel rotation. In other words, the staff members working in the ward at all four time-points were not constant and slightly changed. This was the first installation of the CREW and survey in Japan.

### 2.1. The CREW Program Process

The CREW program was installed over six months, and 18 sessions (3 sessions a month) were held in the ward. Sessions were held during working hours for about 30 min per session and were facilitated by the three ward nurses trained by the research team before installing the CREW program. Staff participation in the CREW program was not mandatory, but the session was announced during the morning conference, and CREW facilitators in the ward asked staff members working on the shift to participate in the CREW program. In each session, the CREW toolkit (e.g., “How do we show respect to one another here?”) [22] and original content of the ward (e.g., “My recommended place.” and “What I want to do this winter?”) were used. In addition, original exercise sheets of the ward were adapted to assess some of the challenges being faced in the ward (e.g., “What kind of ward climate improve safety management?” and “What is your ideal for a conference?”). Participants shared their thoughts with other participants during CREW sessions, with a focus on interpersonal relationships. Consequently, local and open dialog about the civility of individuals and correlated civility was prioritized. Research team members supported CREW facilitators in planning each CREW session and recorded the number of participants at each CREW session and their professions.

### 2.2. Survey Procedure

**Figure 1:**
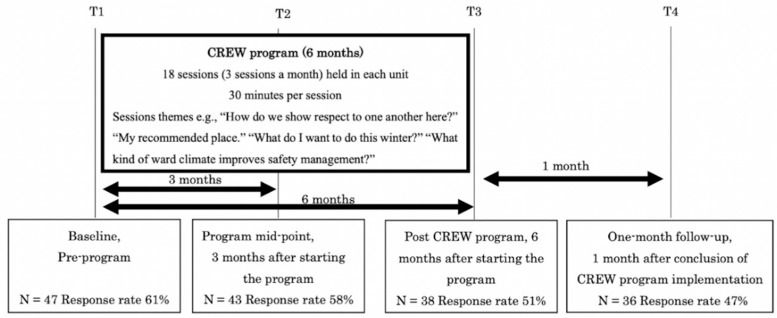
Survey process and CREW process relationships and timeline.

The outcomes of this study were social climate and the individual work engagement of all the staff members working in the ward assessed by the self-administered questionnaire survey. Because the CREW program was intended to change the workplace climate, we observed the effect on all staff members who worked in the ward that installed the CREW program. Therefore, the survey respondents were recruited from all staff members working in the ward regardless of their participation in the CREW program. Participation in the study was completely voluntary, and anonymity and confidentiality were assured. Questionnaires were provided at baseline (T1, August 2014), at the midpoint (T2, three months after starting the program), after the last session of the CREW program as a post-program (T3, six months after baseline), and one month after the end of the CREW program as a follow-up (T4, March 2015). Over six months, staff members in the ward fluctuated over time. Study purposes and procedures were explained to all staff members. Questionnaires were distributed at the workplace through the sub-chiefs of the ward and collected in sealed envelopes via a collection box. Figure 1 shows the study flow.

### 2.3. Outcome Measurement and Assessment

The questionnaire included an assessment of the ward’s social climate, individual work engagement, and other demographic variables. The following surveys measured the social climate: the Essen climate evaluation schema (EssenCES) and the CREW civility scale (civility). Work engagement level was measured using the Utrecht work engagement scale (UWES).

#### 2.3.1. Social Climate

##### EssenCES

To assess the social climate at each time point, we used a subscale of the Japanese version of the EssenCES [40], originally developed by Schalast et al. [41]. The EssenCES is a fifteen-item questionnaire consisting of three subscales with five items for each subscale. The subscales include: (1) therapeutic hold (the extent to which the climate is perceived as supportive of patients’ therapeutic needs, e.g., “Staff take a personal interest in the progress of patients”; “Staff members spend a considerable amount of time to deal with patients”); (2) experienced safety (the level of perceived tension and threat of aggression and violence); and (3) patient cohesion and mutual support (perceived presence of mutual support of a kind typically seen in therapeutic communities). We used only the therapeutic hold subscale in this study because this subscale focuses on the social climate. Items were scored on a five-point Likert-type scale ranging from 0 (not at all) to 4 (very much). Scores ranged from 0 to 20. High scores indicated a positive social climate. The Japanese version of the therapeutic hold subscale has good reliability and validity (Cronbach’s α = 0.77) [42].

##### CREW Civility Scale (Civility)

To assess the civility levels at each time point, we used the Japanese version of the CREW civility scale [43], which was originally developed by Osatuke et al. [22]. The scale consists of eight items designed to measure perceptions about workplace civility within a workgroup and across an organization (e.g., “People treat each other with respect in my workgroup”; “Disputes or conflicts are resolved fairly in my workgroup”; “The people I work with take a personal interest in me”; “Differences among individuals are respected and valued in my workgroup”). The items were rated on a five-point Likert-type scale ranging from 1 (strongly disagree) to 5 (strongly agree). Scores ranged from 0 to 5. Higher scores indicated a greater civility level. Score changes were, therefore, interpreted as workgroup-level changes in a climate of civility. For each respondent, a single index of workgroup civility was calculated as an average of the eight items utilized in this study. Good reliability of the Japanese version has been reported (Cronbach’s α = 0.93) [43].

#### 2.3.2. Work Engagement

##### Utrecht Work Engagement Scale (UWES)

Work engagement at each time point was assessed using the nine-item Japanese version of the Utrecht work engagement scale (UWES) [44], which was originally developed by Schaufeli et al. [11]. UWES consists of three subscales containing nine items (i.e., vigor, dedication, and absorption). Items were scored on a seven-point Likert-type scale ranging from 0 = never to 6 = always (every day). Sample items are, “At my job, I feel strong and vigorous” (vigor), “I am enthusiastic about my job” (dedication), and “I am immersed in my work” (absorption). A total score was calculated using all nine items. Scores ranged from 0 to 6. Higher scores indicated greater work engagement. In this study, we calculated scores by averaging individual item scores. Good reliability and validity of the Japanese version have been confirmed (Cronbach’s α = 0.92) [44].

#### 2.3.3. Demographic Characteristics and Freeform Comments

Demographic characteristics were also collected through the self-administered questionnaire. Items included age, gender, work-related characteristics, occupation, employment status (full-time job or part-time job), and years of experience on the job. Age was classified into five groups: 60 years old or older, 50–59 years old, 40–49 years old, 30–39 years old, and 20–29 years old. The occupation was classified into three groups: nurse, a medical doctor (psychiatrists and residents), and other (psychiatric social workers, occupational therapists, clinical psychologists, pharmacists, cleaning staff, nursing assistants, and medical clerk). In addition, we provided a blank space where respondents could write comments related to the job or the CREW program.

### 2.4. Statistical Analysis

Levene’s test was conducted to assess baseline differences in nonparametric characteristics of staff members working in the ward in four time-points. One-way analysis of variance (ANOVA) was used to assess the differences in the outcome scores between time points. The level of significance was 0.05 (two-tailed). Means and standard deviations (SD) were reported for each outcome variable at baseline (T1), midpoint (T2), after the program (T3), and one-month follow-up (T4). Effect sizes (Cohen’s d) [45] were also calculated from baseline (T1) to midpoint (T2), after the program (T3), and for one-month follow-up (T4). Effect sizes greater than 0.8, 0.5, and 0.2 were considered large, medium, and small, respectively [45]. We followed methodological guidelines [46] and imputed missing values using the regression imputation with relevant variables as covariates for each missing value if the respondents answered more than two-thirds of the scale items. All statistical analyses were conducted with IBM SPSS Statistics version 24 for Windows.

### 2.5. Ethics

The Ethical Committee of the University of Tokyo approved the aims and procedures of this study (No. 10560).

To ensure all ethical standards were met, the subjects were informed in writing about the purpose and methods of this study and data storage and privacy protection methods. Respondents sealed the completed questionnaire in an envelope individually to maintain confidentiality. Staff members were also informed that the participation in the survey was voluntary, and nonparticipants would not be disadvantaged. Respondents indicated their consent by responding to the questionnaire.

## 3. Results

At T1 of the data collection, 77 staff members belonged to the ward, and 47 staff members (61%) answered the self-administered questionnaire. Due to the staff rotation of the residents and nurses in the hospital, the staff members differed and changed over time and at each time point. Overall, 43 out of 74 (58%), 38 out of 74 (51%), and 36 out of 76 (47%) answered the questionnaires at T2, T3, and T4, respectively. Demographic characteristics for participants by each time point are presented in Table 1. Most respondents were female and nurses. No significant differences existed in the demographic characteristics between each time point (*p* > 0.05). The respondents’ descriptions by specific occupation across the four time-points are included in the supplementary file (Table S1).

The average number of participants per CREW session was 9.7, and most participants were nurses, with an average number of 7.8 nurses per session. Besides nurses, other participants included medical doctors, psychiatric social workers, occupational therapists, clinical psychologists, and pharmacists.

The effects of the CREW program on the social climate and work engagement for all staff members and by occupation are shown in Table 2. No statistically significant differences emerged in any of the outcome scores between baseline and T2, T3, and T4.

The effect sizes on EssenCES were small for all staff members from baseline to T3. Among medical doctors, the effect sizes were large on both EssenCES and civility of 0.76 (95% CI: −0.12 to 1.64) and 0.79 (95% CI: −0.09 to 1.67), respectively, from baseline to T3. Among nurses, the effect sizes were medium on both EssenCES and UWES of 0.57 (95% CI: −0.10 to 1.24) and 0.56 (95% CI: −0.11 to 1.23), respectively from baseline to T3. In summary, the effects were non-significant regardless of the effect sizes.

Some respondents commented on the social climate in the blank space provided, stating, for example, “Our workplace climate has become positive”, “Staff cooperation was strengthened in our ward”, “Felt the sense of unity among the staff”. Negative comments about the CREW program included, “It was tough to participate in CREW sessions during working hours because we are busy”.

## 4. Discussion

The purpose of this study was to examine the effects of the CREW program on the social climate and work engagement among psychiatric ward staff at a Japanese teaching hospital. The hypothesis was that social climate and work engagement assessed by the staff members working in a Japanese hospital would be better after the CREW program installation than the time-point before the program was installed.

ANOVA results were not significant for all outcome variables. The effect size for the social climate assessed by EssenCES was small, and the effect sizes for the civility scale and work engagement were even smaller. The confidence intervals for all effect sizes included zero. This means that we cannot claim that social climate or work engagement, as assessed by the ward staff members before and after the CREW program installation in the ward, changed over time. Furthermore, in this study, the CREW program’s effects were not sustained one month after its installation at T4. In a previous study, the CREW program showed significant improvement in civility norms (i.e., civility, incivility, and respect) and work-related outcomes (i.e., job satisfaction, organizational commitment, management trust, and job burnout) [22,23,24], and the improvements were sustained one-year post-implementation [23,24].

In this study, no changes were observed for several reasons. First, as mentioned in the participants’ comments, CREW sessions may have burdened busy healthcare staff, which could have curtailed the effect of the CREW program. Second, the number of participants other than nurses in the CREW program was modest, and the healthcare staff at the ward may not have been familiar with the CREW program. Third, social climate and work engagement baseline scores in this study were slightly higher compared to the scores reported for Japanese nurses. The mean of UWES was 2.2 (SD ± 1.0) for Japanese nurses (n = 300, including psychiatric nurses) [47], and the mean of EssenCES was 13.38 (SD not shown) for the Japanese psychiatric nurses (n = 227) [42]. As such, the CREW program might not have been sufficiently intense to improve work engagement among workers, who already had high work engagement and EssenCES at baseline. The mean civility score was 3.94 (SD ± 0.72) among Japanese employees (n = 2191), and it did not differ across respondents.

In terms of outcome results by occupation, although most of the participants in the CREW session were nurses, the effect sizes on the perceived civility and social climate in the ward were larger for medical doctors than for nurses. This result may suggest a crossover effect. Previous studies have indicated that climate change is often contagious in the most positive sense within the organization, likely causing a “civility spiral” [22,23]. Therefore, even staff members who had not participated in the CREW program session may perceive the climate change in their workplace and perceive the effect of the CREW program implementation on the ward (i.e., a spillover effect) [22]. Positive encounters with respect and civility are associated with more direct access to emotional support and increased energy from colleagues’ crossover effects or spillover effects.

To the best of our knowledge, this study is the first to report the results of the installation of the CREW program in an Asian setting. We also reported participants’ comments to an open-ended question, supplementing the quantitative data. The study result can form the basis for further CREW study in Japan. Furthermore, evaluating the effects of the CREW program from the perspective of program session participants, including their perceptions of the program itself and its effect on the individual participants, appears useful for subsequent research, both qualitative and quantitative.

Although we did not find significant score improvements, the CREW program was implemented for six months without interruption in the ward, and the participants’ comments revealed that the ward climate changed for the better, and they feel that the cooperation among the staff increased. In addition, after the study period, the nurse manager, who proposed the installation of the CREW program, retired; nevertheless, the CREW program session has been implemented in the ward on an ongoing basis, although less frequently (e.g., once a month). This suggests that the staff members in the ward believed that the program had some positive effect, and its implementation may be of value to staff members working in hospitals. The CREW program installed in the Japanese psychiatric ward in this study did not show the same effect as in the previous studies. However, larger studies are needed to evaluate the effectiveness of this program and other interventions that could effectively improve social climate change and work engagement in healthcare settings. Future studies could interview the ward staff and carefully investigate the elements of the CREW program that affect the Japanese psychiatric ward workers.

## 5. Limitations

This study has several limitations. First, the follow-up survey was conducted at the end of the fiscal year, which is an extremely busy period in healthcare settings, and the influence of this situation could not be verified. Second, survey response rates were 47 to 61 percent among the staff members working in the ward at each time point. It is possible that the staff who responded to the survey were supportive and showed a more positive attitude towards nonrespondents. Finally, the CREW program is intended to change the workplace climate. Thus, we intended to measure the CREW program’s effect on all staff members, who worked in the ward that installed the program, regardless of whether they participated in the CREW sessions, and we did not examine the effects on people who did not answer the survey. In addition, we did not investigate the participation status of respondents. Therefore, how exposure to the CREW program affected healthcare staff is unclear. In this pilot study, it might have been better to consider other ways to assess the social climate in the ward (e.g., observer evaluation) and conduct an evaluation based on the participation status.

## 6. Conclusions

This study did not demonstrate statistically significant differences in social climate and work engagement among healthcare workers between before and after the installation of the CREW program in a Japanese psychiatric ward. However, the effect size was small to large. Overall, this pilot study showed that installing the CREW program in a psychiatric ward at a single hospital was feasible and well-received. We believe that presenting the effect sizes of our study could inform further research on social climate and/or work engagement in workplaces. A larger study should be conducted in the future to evaluate the effect of this program.

## Figures and Tables

**Table 1:** Study participant demographics at each time point in a psychiatric ward in a Japanese hospital.

	Baseline (T1)		Midpoint (T2)		Post (T3)		One-Month Post (T4)		*p*
	n = 47		n = 43		n = 38		n = 36		
	n (%)	Mean (SD)	n (%)	Mean (SD)	n (%)	Mean (SD)	n (%)	Mean (SD)	
Gender									0.549
Male	11 (23.4)		6 (14.0)		10 (26.3)		7 (19.4)		
Female	35 (74.5)		37 (86.0)		28 (73.7)		26 (72.2)		
Not provided	1 (2.1)		-		-		3 (8.3)		
Age									0.901
20–29	19 (40.4)		18 (41.9)		17 (44.7)		15 (41.7)		
30–39	14 (29.8)		10 (23.3)		10 (26.3)		9 (25)		
40–49	8 (17.0)		7 (16.3)		4 (10.5)		7 (19.4)		
50–59	3 (6.4)		4 (9.3)		4 (10.5)		3 (8.3)		
Over 60	3 (6.4)		3 (7.0)		2 (5.3)		-		
Not provided	-		1 (2.0)		1 (2.6)		2 (5.6)		
Job									0.837
Nurse	22 (46.8)		25 (58.1)		17 (44.7)		19 (52.8)		
Medical doctor	13 (27.7)		9 (20.9)		11 (28.9)		9 (25.0)		
Others	10 (21.3)		9 (20.9)		8 (21.1)		6 (16.7)		
Not provided	2 (4.3)		-		2 (5.3)		2 (5.6)		
Employment status									0.446
Full-time	38 (80.9)		34 (79.1)		25 (65.8)		26 (72.2)		
Part-time	9 (19.1)		8 (18.6)		12 (31.6)		7 (19.4)		
Not provided	-		1 (2.3)		1 (2.6)		3 (8.3)		
Years of experience in job									0.796
All		4.2 (5.1)		8 (7.3)		7.1 (6.5)		7.9 (6.5)	
Nurse		7.9 (8.1)		8.1 (7.7)		8.6 (8.5)		8.2 (6.7)	
Medical doctor		8.8 (7.5)		7.1 (8.3)		4.9 (5.4)		7.9 (8.1)	
Others		9.7 (9.0)		8.5 (5.6)		7.1 (2.5)		7 (2.7)	

Note: T1 (baseline = August 2014), T2 (at the midpoint, three months after starting the program), T3 (post-program after the last session of the CREW program conducted six months after the baseline), and T4 (one-month follow-up after the end of the CREW program = March 2015). Others: psychiatric social workers, occupational therapists, clinical psychologists, pharmacists, cleaning staff, nursing assistants, and medical clerks.

**Table 2:** Scores on three outcome variables and results analysis of variance by time.

		Baseline (T1)			Mid-Survey (T2)			Post-Survey (T3)			1-Month Follow-Up Survey (T4)					Cohen’s *d* (95% CI; LL–UL)		
Variables (Range Potential)		**n**	**Mean**	**SD**	**n**	**Mean**	**SD**	**n**	**Mean**	**SD**	**n**	**Mean**	**SD**	***F***	***p***	**T2-T1**	**T3-T1**	**T4-T1**
EssenCES	All *	46	14.0	2.6	41	14.3	2.1	37	14.9	2.5	36	13.8	2.4	1.65	0.17	0.13 (−0.30–0.55)	0.35 (−0.09–0.79)	−0.08 (−0.52–0.36)
(0–20)	Nurse	22	13.5	3.0	25	14.2	2.1	17	15.1	2.5	19	14.3	2.0	1.32	0.28	0.27 (−0.32–0.86)	0.57 (−0.10–1.24)	0.31 (−0.33–0.95)
	Medical doctors	13	13.8	2.2	9	13.8	2.6	11	15.5	2.3	9	13.1	3.4	1.54	0.22	0.0 (−0.90–0.90)	0.76 (−0.12–1.64)	−0.18 (−1.09–0.72)
	Others	9	15.1	1.9	7	15.1	1.3	7	14.7	2.5	6	13.7	2.0	0.80	0.51	0.0 (−1.08–1.08)	−0.18 (−1.27–0.90)	−0.73 (−1.90–0.45)
Civility	All *	46	3.9	0.5	39	3.9	0.4	36	3.9	0.5	34	3.8	0.4	0.40	0.76	0.0 (−0.43–0.43)	0.0 (−0.44–0.44)	−0.01 (−0.46–0.44)
(0–5)	Nurse	22	3.9	0.6	25	3.9	0.4	17	3.9	0.4	19	3.9	0.4	0.01	0.99	0.0 (−0.59–0.59)	0.0 (−0.65–0.65)	−0.06 (−0.69–0.58)
	Medical doctors	13	3.8	0.5	9	4.1	0.4	11	4.2	0.5	8	3.7	0.3	2.35	0.09	0.63 (−0.30–1.55)	0.79 (−0.09–1.67)	−0.18 (−1.12–0.76)
	Others	9	4.0	0.5	5	3.8	0.5	6	3.6	0.4	5	3.8	0.5	0.66	0.59	−0.41 (−1.63–0.82)	−0.84 (−2.03–0.34)	−0.39 (−1.61–0.84)
UWES	All *	47	2.9	1.1	42	2.8	0.9	38	3.0	1.0	35	2.6	1.2	1.11	0.44	−0.10 (−0.52–0.32)	0.09 (−0.34–0.53)	−0.25 (−0.69–0.2)
(0–5)	Nurse	22	2.4	0.8	25	2.7	0.9	17	2.9	1.0	19	2.6	1.1	0.87	0.46	0.35 (−0.24–0.94)	0.56 (−0.11–1.23)	0.24 (−0.4–0.88)
	Medical doctors	13	3.1	1.0	9	3.3	0.9	11	3.3	1.3	9	2.7	1.4	0.68	0.57	0.21 (−0.70–1.12)	0.17 (−0.68–1.03)	−0.37 (−1.28–0.54)
	Others	10	3.5	1.4	8	2.8	1.0	8	3.2	0.7	6	3.1	0.6	0.60	0.62	−0.71 (−1.75–0.32)	−0.34 (−1.35–0.60)	−0.48 (−1.6–0.64)

Note: one-way analysis of variance (ANOVA) was used to compare the means across T1 (baseline = August 2014), T2 (at the midpoint, three months after starting the program), T3 (post-program after the last session of the CREW program conducted six months after the baseline), and T4 (one-month follow-up after the end of the CREW program = March 2015). EssenCES: Essen climate evaluation schema—therapeutic hold (range actual: 7–20, 9–18, 10–20 and 7–20 at T1, T2, T3 and T4, respectively), civility: The Japanese version CREW civility survey (range actual: 2.5–5.0, 3.0–5.0, 2.88–5.0 and 3.0–5.0, at T1, T2, T3 and T4, respectively), UWES: Utrecht work engagement scale (range actual: 0.3–6.0, 0–5.2, 0.7–5.3 and 0–4.5 at T1, T2, T3 and T4, respectively). CI = confidence interval; LL = lower limit; UL = upper limit. All *: data for the group of people who did not provide their job is also included in “All”. Others: psychiatric social workers, occupational therapists, clinical psychologists, pharmacists, cleaning staff, nursing assistants, and medical clerks.

## Data Availability

The data used for this present study may be obtained from the corresponding author via e-mail.

## Supplementary Materials

The following are available online at https://www.mdpi.com/article/10.3390/nursrep11020031/s1, Table S1: Study Participant Demographics at Each Time Point by Occupation.
