# Supplementary material for: The Effects of the Civility, Respect, and Engagement in the Workplace (CREW) Program on Social Climate and Work Engagement in a Psychiatric Ward in Japan: A Pilot Study
Source: Nurs Rep. 2021 May 1;11(2):320–30. doi: 10.3390/nursrep11020031 (PMC8608135; doi:10.3390/nursrep11020031)
Supplement: Supplementary file 1 [file nursrep-11-00031-s001.zip › nursrep-1129802-supplementary.pdf]

Table S1. Study Participant Demographics at Each Time Point by Occupation.

	Baseline (T1) n = 47		Mid - point (T2) n = 43		Post (T3) n = 38		One-month post (T4) n = 36	
	n (%)	mean (SD)	n (%)	mean (SD)	n (%)	mean (SD)	n (%)	mean (SD)
**Nurse**	22 (46.8)		25 (58.1)		17 (44.7)		19 (52.8)	
Gender								
Male	1 (4.5)		1 (10.0)		1 (10.0)		1 (10.0)	
Female	20 (91.0)		24 (90.0)		16 (90.0)		18 (90.0)	
Not provided	1 (4.5)		-		-		-	
Age								
20-29	13 (59.1)		12 (48.0)		8 (47.1)		9 (47.4)	
30-39	4 (18.2)		6 (24.0)		4 (23.5)		5 (26.3)	
40-49	4 (18.2)		4 (16.0)		2 (11.8)		4 (21.1)	
50-59	1 (4.5)		2 (8.0)		2 (11.8)		1 (5.2)	
Over 60	-		-		-		-	
Not provided	-		1 (4.0)		1 (5.8)		-	
Employment status								
Full-Time	22 (100)		25 (100)		17 (100)		19 (100)	
Part-Time	-		-		-		-	
Not provided	-		-		-		-	
Years of experience in job		7.9 (8.1)		8.1 (7.7)		8.6 (8.5)		8.2 (6.7)
**Medical Doctor**	13 (27.7)		9 (20.9)		11 (28.9)		9 (25)	
Gender								
Male	9 (69.2)		6 (66.7)		9 (81.8)		5 (55.6)	
Female	4 (30.8)		3 (33.3)		2 (18.2)		4 (44.4)	
Not provided	-		-		-		-	
Age								
20-29	3 (23.0)		4 (44.5)		6 (54.5)		4 (44.5)	
30-39	7 (54.0)		3 (33.3)		4 (36.4)		3 (33.3)	
40-49	3 (23.0)		2 (22.2)		1 (9.1)		2 (22.2)	
50-59	-		-		-		-	
Over 60	-		-		-		-	
Not provided	-		-		-		-	
Employment status								
Full-Time	8 (61.5)		5 (55.6)		3 (27.3)		6 (66.7)	
Part-Time	5 (38.5)		4 (44.4)		8 (72.7)		3 (33.3)	
Not provided	-		-		-		-	
Years of experience in job		8.8 (7.5)		7.1 (8.3)		4.9 (5.4)		7.9 (8.1)
**Others**	10 (21.3)		9 (20.9)		8 (21.1)		6 (16.7)	
Gender								
Male	1 (10.0)		-		-		-	
Female	9 (9.0)		9 (100)		8 (100)		6 ()	
Not provided	-		-		-		-	
Age								
20-29	2 (20.0)		2 (22.2)		2 (25.0)		2 (33.3)	
30-39	2 (20.0)		1 (11.1)		1 (12.5)		1 (16.7)	
40-49	1 (10.0)		1 (11.1)		1 (12.5)		1 (16.7)	
50-59	2 (20.0)		2 (22.2)		2 (25.0)		2 (33.3)	
Over 60	3 (10.0)		3 (33.4)		2 (25.0)		-	
Not provided	-		-		-		-	

Employment status								
Full-Time	6 (60.0)		3 (33.3)		3 (37.5)		3 (50.0)	
Part-Time	4 (40.0)		5 (55.6)		4 (50.0)		3 (50.0)	
Not provided	-		1 (11.1)		1 (12.5)		-	
Years of experience in job		9.7 (9.0)		8.5 (5.6)		7.1 (2.5)		7 (2.7)
**Not provided**	2 (4.2)		-		2 (5.3)		-	
Gender								
Male	-		-		-		-	
Female	2 (100.0)		-		2 (100.0)		-	
Not provided	-		-		-		-	
Age								
20-29	-		-		-		-	
30-39	1 (50.0)		-		1 (50.0)		-	
40-49	-		-		1 (50.0)		-	
50-59	1 (50.0)		-		-		-	
Over 60	-		-		-		-	
Not provided	-		-		-		-	
Employment status								
Full-Time	2 (100.0)		-		2 (100.0)		-	
Part-Time	-		-		-		-	
Not provided	-		-		-		-	
Years of experience in job		6.0 (1.5)		-		5.9 (1.0)		-

Note. T1(baseline = August 2014), T2 (at mid-point, three months after starting the program), T3 (post-program after the last session of the CREW program conducted six months after the baseline), and T4 (one-month follow-up after the end of the CREW program = March 2015). Others: psychiatric social workers, occupational therapists, clinical psychologists, pharmacists, cleaning staff, nursing assistants, and medical clerks.
